# Contact Heterogeneity and Phylodynamics: How Contact Networks Shape Parasite Evolutionary Trees

**DOI:** 10.1155/2011/238743

**Published:** 2010-12-01

**Authors:** Eamon B. O'Dea, Claus O. Wilke

**Affiliations:** ^1^Section of Integrative Biology, The University of Texas at Austin, Austin, TX 78712, USA; ^2^Center for Computational Biology and Bioinformatics, The University of Texas at Austin, Austin, TX 78712, USA; ^3^Institute for Cellular and Molecular Biology, The University of Texas at Austin, Austin, TX 78712, USA

## Abstract

The inference of population dynamics from molecular sequence data is becoming an important new method for the surveillance of infectious diseases. Here, we examine how heterogeneity in contact shapes the genealogies of parasitic agents. Using extensive simulations, we find that contact heterogeneity can have a strong effect on how the structure of genealogies reflects epidemiologically relevant quantities such as the proportion of a population that is infected. Comparing the simulations to BEAST reconstructions, we also find that contact heterogeneity can increase the number of sequence isolates required to estimate these quantities over the course of an epidemic. Our results suggest that data about contact-network structure will be required in addition to sequence data for accurate estimation of a parasitic agent's genealogy. We conclude that network models will be important for progress in this area.

## 1. Introduction

 Epidemiology is a data-driven field, and it is currently being infused at an increasing rate with molecular sequence data. This new and growing data source has led to a call for multi-level models of the relationship between sequence data and infectious disease dynamics [1, 2], dubbed phylodynamic models.

By allowing for additional data to be used and integrated, phylodynamic modeling may lead to improvements in the accuracy and quality of the surveillance of infectious diseases. For example, the number of norovirus outbreaks reported increased in 2002. It was not clear, however, whether the higher reported numbers were a sign of more outbreaks or more frequent reporting of outbreaks. Case-reporting bias does not affect molecular data, however. So coalescent analysis of molecular data [3] provided a valuable and largely independent line of evidence that the increase in outbreaks was real. Of course, coalescent analysis will have its own biases, and here we examine those that result from host heterogeneity in contact.

To model heterogeneity in contact, we represent individuals in a population as nodes, and we represent the potential for two hosts to infect each other as an edge that links two nodes. Researchers call the resulting networks contact networks. Contact-network structure necessarily affects the genealogy of any replicating infectious agent that is spreading through a host population. In this paper, we use the term parasite to refer to all such infectious agents, including bacteria and viruses. The genealogy of these parasites must fit inside the tree of infections that forms as the parasite spreads from host to host, and this tree of infections must fit inside the host population's contact network. While more elaborate elements of contact-network structure may be important, we here focus simply on variation in the number of edges coming out of nodes, which corresponds to heterogeneity in contact rates.

Contact heterogeneity has often not been discussed as a possible bias in coalescent analyses (e.g., [4–6]). Researchers performing coalescent analyses have considered contact heterogeneity in a variety of other ways. Hughes et al. [7] linked it to the phylogenetic clustering of sequence isolates. Biek et al. [8] mentioned that it may have contributed to changes in an estimation of *R*_0_ (the expected number of new cases that a single case produces in a susceptible population). Nakano et al. [9] discussed how iatrogenic transmission may have been an important type of transmission in the spread of hepatitis C. Bennett et al. [10] pointed out that population-size estimates from coalescent analyses are more accurately interpreted as ratios of population size to reproductive variance. But researchers have rarely quantitatively considered how contact heterogeneity might be directly influencing the results of their coalescent analyses. Volz et al. [11] did account for contact heterogeneity in their coalescent model with a saturation parameter, but this application does not provide a general illustration of how contact-network structure can affect genealogies.

Our primary goal here is to assess how contact heterogeneity affects the relationship between coalescent reconstructions and the reality of parasite population dynamics. First, we build contact networks with different levels of heterogeneity. Then, we simulate the spread of parasites through the networks, generating epidemic dynamics and a genealogy of the parasite with each simulation. Then, we use the BEAST software package [12] to produce Bayesian skyride [13] reconstructions of parasite population dynamics based on the simulated genealogies. We also use the framework of Volz et al. [11] to predict the skyride reconstructions based on the simulated epidemic dynamics. We explain how the contact-network structure affects the epidemic dynamics that, in turn, affect the predicted reconstructions. The close agreement between the predicted skyrides and the skyride reconstructions validates this explanation. We also examine how much of the simulated genealogy the skyride reconstruction requires as input in order to produce a reconstruction that agrees with the theoretical prediction.

## 2. Materials and Methods

 We simulated infectious disease progression on networks. The nodes of the networks represented hosts and had states of being susceptible, infectious, or recovered. The edges of the network determined the set of possible transmission events; infectious hosts transmitted infection across edges shared with susceptible hosts until the infectious hosts recovered. The number of nodes in the network was kept at 10,000, and the mean degree (degree is the number of edges coming out of a node) was kept at 4. The networks were built to be either regular, meaning that all nodes have the same degree, or with degree distributions sampled from Poisson, exponential, or Pareto distributions. The minimum degree in the Pareto networks was 1. The regular networks served as models with zero heterogeneity, Poisson networks as models with heterogeneity similar to a Poisson process, exponential networks as models with heterogeneity similar to a variety of social networks [14], and Pareto networks (scale-free networks) as models with the extreme levels of heterogeneity that might be found in sexual contact networks [15]. We used the Erdös-Rényi algorithm [16] to generate Poisson networks and an edge-shuffling algorithm [17] to generate the regular, exponential, and Pareto networks.

We simulated epidemics and genealogies in continuous time using a method based on the Stochastic Simulation Algorithm [18, 19]. Epidemics began with one node infectious and the rest of the nodes being susceptible. Infectious nodes recovered at a set rate and transmitted infection to susceptible neighbors (nodes sharing an edge) at a set rate. We drew the time to the next event from an exponential distribution with a rate equal to the sum of the rates of all possible events. We then selected an event with probability proportional to its rate, updated the state of the network accordingly, and drew the time until the next event. This process was iterated until either the time evolution of the epidemic reached a set time point or no more events were possible.

Simulation source code is available from the authors upon request. The code made use of the GNU scientific library [20, version 1.13+dsfg-1] to generate random numbers and the igraph library [21, version 0.5.3-6] to construct networks.

The output of a simulation included a time series of prevalence, that is, the count of infected nodes (given a fixed population of 10,000 nodes), and incidence, that is, the sum of the rates of all possible transmissions. Simulations also generated infection trees in which each transmission was a bifurcating node, each recovery was a terminal node, and branch lengths were equal to the time between events. We sampled from the full infection trees to generate the trees for input in the skyride coalescent analyses. We sampled by selecting a set of nodes uniformly at random from the full infection tree to become tip branches of an infection subtree. To generate the subtree, we cut the branches of the full infection tree at the subset of randomly selected nodes that had no descendants in the set of randomly selected nodes, and we pruned off any paths that did not terminate in this subset of nodes.

Using the sampled infection trees as genealogies, we obtained a posterior distribution for the skyride population sizes with the time-aware method of Minin et al. [13], implemented in BEAST [12, version 1.5.4]. The MCMC chain lengths were 100,000 states, and every 10th state was written to a log file. We discarded the first 10,000 states as burn in. In all cases, effective sample sizes were well above 200. Thus, convergence had occurred. Examples of BEAST XML input files are available from the authors upon request.

Using the posterior skyride population-size distributions, we obtained the skyride trajectories with Tracer [22, version 1.5]. Using the framework of Volz et al. [11], we calculated a predicted skyride as described next in the Results.

To plot time series from different stochastic simulations on a common time scale, we used the time at which growth became nearly deterministic in each simulation as time zero for that simulation.

## 3. Results

### 3.1. Theory

 Coalescent theory is an area of population genetics that models the structure of genealogies backward in time from a set of lineages sampled from a large population. A simple coalescent process turns out to be a good model for the genealogies of a wide range of scenarios in population genetics [23]. In the coalescent process, each pair of lineages in the sample coalesces into a common ancestral lineage at a constant rate. When time is measured in units of generations, this rate is the reciprocal of the effective population size. So the rate at which any of the pairs coalesces is equal to the number of pairs of lineages divided by the effective population size.

The skyride uses this simple relationship between effective population size and the expected time before coalescence to estimate population size from the length of intracoalescent intervals in a genealogy. The median of a skyride reconstruction *y*_rec_ at time *t* within an intracoalescent interval is approximately

(1) $$\begin{array}{c} y_{\text{rec}}=N_{e}\tau =\left(\begin{array}{c} n\\ 2 \end{array}\right)u, \end{array}$$

where *N*_*e*_ is the effective population size, *τ* is the generation time, $\left(\begin{array}{c} n\\ 2 \end{array}\right)$ is the average number of pairs of lineages in the sample within the intracoalescent interval, and *u* is the length of the intracoalescent interval.

Predicting a skyride from the dynamics of an epidemic model is simply a matter of calculating the rate at which a pair of lineages will coalesce, that is, the rate at which two chains of infection merge into a single chain. Volz et al. [11] have described how coalescence rates follow from prevalence and incidence. Prevalence, given a fixed population size, refers to the count of cases of infection, and so we denote it by *I*. Incidence refers to the rate at which new cases are occurring, and so we denote it by *r*_*i*_. The rate of coalescence of a single pair of cases is

(2) $$\begin{array}{c} r_{i}P, \end{array}$$

where *P* is the probability that we can trace a particular pair of cases back to a single case before the last transmission event. We have

(3) $$\begin{array}{c} P=1/\left(\begin{array}{c} I\\ 2 \end{array}\right), \end{array}$$

making the approximation that the last transmission event was equally likely to have taken place between any pair of current cases. Therefore, the predicted skyride *y*_pred_ satisfies

(4) $$\begin{array}{c} y_{\text{pred}}=\frac{1}{r_{i}P}=\left(\begin{array}{c} I\\ 2 \end{array}\right)/r_{i}. \end{array}$$

The similarity of (4) and (1) reflects the similarity of the coalescent process to the transmission process in a continuous-time epidemic model. *N*_*e*_ and *τ*, however, are often considered as parameters of a discrete-time population model that has nonoverlapping generations. The coalescent process describes the genealogy in such a model when we sample a small fraction of the lineages in a population. So how do we interpret *N*_*e*_ and *τ* in the terms of a continuous-time epidemic model that has overlapping generations? Following Frost and Volz [24] and the general theory of Wakeley and Sargsyan [25], we say that generation time *τ* is equal to the expected time before an infected individual transmits infection:

(5) $$\begin{array}{c} \begin{array}{c} \tau =\frac{I}{r_{i}}. \end{array} \end{array}$$

Then from (1) and (4) and *y*_rec_ = *y*_pred_, we have

(6) $$\begin{array}{c} N_{e}=\frac{I-1}{2}\approx \frac{I}{2}. \end{array}$$

### 3.2. Simulation

**Figure 1:**
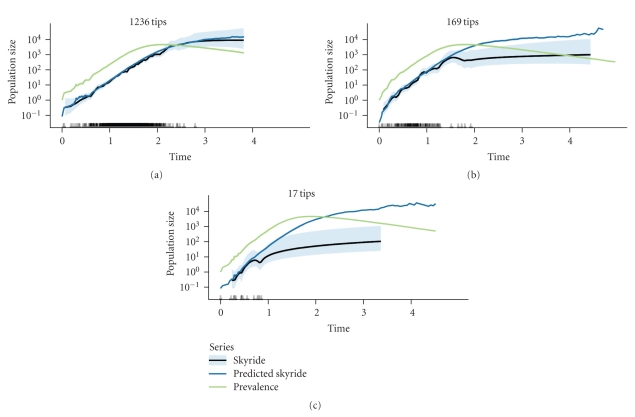
Low levels of proportional sampling may prevent accurate reconstruction of prevalence during and after the epidemic peak. We consider reconstruction to be accurate when the skyride and the predicted skyride match. The light-blue ribbons are the middle 95% of the posterior density of the skyride reconstruction. The small bars on the *x*-axis represent the times of coalescent events in the sampled genealogy. Labels above the Panels indicate the number of tips in the sampled genealogy. Parameters: contact-network size = 10,000, Poisson degree distribution with mean = 4, transmission rate = 2, recovery rate = 1, proportion of nodes sampled = {0.1, 0.01, 0.001} ((a), (b), and (c)).

 To determine the effect of sampling on the ability of the skyride to reconstruct prevalence history, we simulated genealogies and pruned off a variable number of branches from the genealogies. We found that small amounts of pruning rapidly reduced the number of coalescent events in the sampled genealogy that occurred in the peak and late phases of the epidemic, thereby restricting accurate reconstruction to the early phase of the epidemic (Figure 1). 

**Figure 2:**
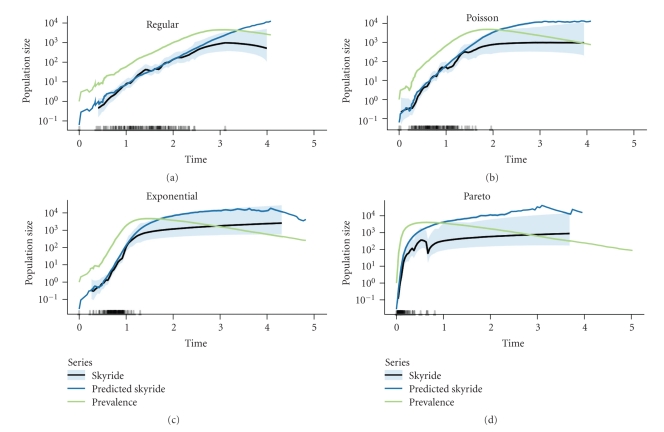
Contact heterogeneity determines the amount of time over which the skyride estimated from the genealogy is informative of the skyride predicted by prevalence and incidence. Contact heterogeneity also affects the relationship between the skyride and prevalence trajectories. The light-blue ribbons are the middle 95% of the posterior density of the skyride reconstruction. The small bars on the *x*-axis represent the times of coalescent events in the sampled genealogy. Labels above the Panels indicate the approximate degree distribution of the contact networks. The variance of the degree distributions increases from (a) to (d). Parameters: contact-network size = 10,000, degree distribution mean = 4, transmission rate = 2, recovery rate = 1, proportion of nodes sampled = 0.01.

To demonstrate the effect of network structure on the reconstruction of prevalence history, epidemics were simulated on networks with varying heterogeneity. Keeping the extent of sampling equal and increasing heterogeneity compressed the coalescent events in the sampled genealogy into the beginning of the epidemic.  Figure 2 shows a representative example of this general trend that holds across intermediate levels of sampling. Consequently, increasing heterogeneity has a similar effect to reducing the proportion of nodes sampled: the time at which the prediction of the skyride based on prevalence and incidence diverges from the estimated skyride based on the genealogy occurs earlier.

**Figure 3:**
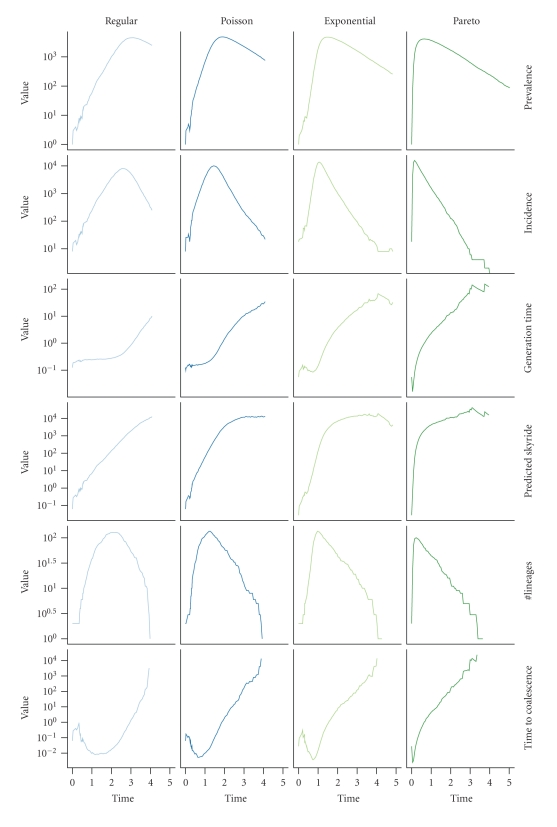
Contact-network structure, infectious disease dynamics, and genealogical structure interact. The ratio of prevalence to incidence is the generation time, which scales prevalence of the predicted skyride (up to a constant factor). Dividing the predicted skyride by the number of pairs of lineages backs out a smoothed expected length of intracoalescent intervals in the genealogy. Panel labels on the top indicate the approximate degree distribution of the contact networks. The variance of the degree distributions increase from left to right. Parameters: contact-network size = 10,000, degree distribution mean = 4, transmission rate = 2, recovery rate = 1, proportion of nodes sampled = 0.01.

Figure 3 shows how differences in the scaling of prevalence of the skyride follows from differences in trajectories of prevalence and incidence. The ratio of prevalence to incidence is the expected time until an infected host transmits infection, and we here define it as the generation time (5). In Figure 3, we see that generation times are at, or quickly reach, a minimum after an epidemic begins and then gradually increase until the epidemic ends. In the regular networks, the decline in the number of susceptible hosts over the course of the epidemic causes this increase to happen. In the other networks, which have hosts of varying degree, infection first moves to the high-degree hosts and then to progressively lower- and lower-degree hosts [26–28]. Because the degree of a host determines how much his/her infection increases incidence, this movement of infection from high- to low-degree hosts translates into generation times being at first shorter and then longer in heterogeneous networks relative to regular networks (Figure 3). 

## 4. Discussion

 The effects of contact heterogeneity can be important in relating the structure of genealogies to infectious disease dynamics (Figure 3). The strength of the effect will vary from system to system, and for some systems other aspects of contact-network structure such as the frequency of short paths [29] and the dynamics of edge formation [30–33] may also be important. More generally, models may also require more detailed models of the course of infection within hosts (including incubation periods, e.g.), the effects of natural selection [34, 35], and other additions before they can make precise predictions in real-world systems.

But are the data requirements of these more complex models feasible? To begin answering this question, we next discuss the implications of obtaining the equivalent of our simulated data from a real-world system.

We knew the true infection tree in our simulations. In typical coalescent analyses of an infectious disease (e.g., [13, 36]), we do not know the true genealogy and so we must infer it along with the dynamics of the effective population size. Although there is a large set of methods for the inference of trees from sequences [37–39], the variety of methods available reflects the difficulty of the task. Additionally, as is well known by practitioners of phylogenetics, substitution rates set fundamental limits on the amount of phylogenetic information that sequences may contain. Sequences with common ancestors that are very recent may not have any polymorphic sites that could suggest the structure of the branching of the tree connecting them. Sequences with common ancestors that are too distant similarly contain little information about the true genealogy [40].

It may be possible to work around the second problem by collecting sequences over time such that there are no branching points in the tree that are too far from every pair of tips. For the first problem, there is simply no information that the sequences alone can provide, and additional knowledge of events in the chain of infection is necessary to determine the infection tree. The panels labeled “Time to coalescence” in Figure 3 show that this additional information is most likely to be needed early in the epidemic and when there is a large amount of variance in the contact network. It is then perhaps fortunate that contact-tracing methods are practiced by many health departments for sexually transmitted diseases (STDs) [41, 42], which are thought to have higher contact heterogeneity than airborne diseases [15]. However, we probably need more widespread practice of contact tracing for large genealogies to be assembled. A recent survey of physicians in the United States [43] found that less than one-third of physicians routinely screen patients for STDs and many physicians relied on patients to notify health departments and partners, and similar surveys in other countries [42, 44, 45] likewise indicate that contact tracing is not a routine in general medical care of STDs.

There also may be a need for contact tracing to establish the genealogy for airborne infections because many airborne transmissions may occur in a single day during which a single strain may be dominant in a host, as the super-spreading events in the 2003 SARS-coronavirus outbreak demonstrated [46]. Contact tracing is also practiced for airborne diseases. It has been used to help contain the SARS-coronavirus outbreak [47], smallpox [48], and tuberculosis [49]. Given that contacts for airborne diseases can be quite transient, it seems that, even with the addition of contact-tracing data, we may generally know less about parasite genealogies for airborne diseases compared to STDs. On the upside, our results suggest that the ability to reconstruct early parts of the epidemic is robust to much pruning of the full genealogy (Figure 1). However, this robustness may depend on our sampling scheme. Using discrete-time simulations, Stack et al. [50] found that the difference between reconstructed prevalence and simulated prevalence depended largely on how the samples were distributed over the course of the epidemic. Also, it is unclear how any of our sampling levels might compare to realistic amounts of contact tracing and molecular data for a specific infectious disease.

In addition to being necessary to fill gaps in molecular data, contact tracing may be necessary because genealogies do not always match infection trees. Such discordance is likely to occur when there is relatively little time between transmissions. When there is little time for a mutant to become fixed between transmissions, the order in which alleles at loci of a sequence appear in transmitting inocula (or sequence isolates) need not match the order in which the alleles appeared in the within-host population. Measures of within-host viral load and sequence diversity may be informative of the chance of such discordance. If populations tend to be large and diverse, then sequence data may be useless for reconstructing the recent details of chains of infection but still useful in reconstructing deeper branches in the tree. Sequence data from diverse within-host populations could also be useful in parameter estimation for coalescent models (e.g., [51]) that include the within-host dynamics of the parasite. Two properties that parasites may have that would help increase the chance that infection trees and genealogies match are a low level of diversity in transmitting inocula (i.e., a strong bottleneck effect at transmission) and reduction of diversity in an incubation period that precedes all transmission.

In our simulations, we also knew the variance of the degree distribution. We do have some data about the structure of contact networks for some systems. We have survey data about human sexual-contact networks (e.g., [52, 53]) and survey data about networks of close, but not sexual, human contacts [54–56]. Researchers have used field data to construct hypothetical contact networks for wildlife and vector-borne diseases (e.g., [57, 58]), and researchers have also used census data to construct hypothetical contact networks for human diseases (e.g., [59, 60]). It seems likely, however, that in the analysis of real sequence data the heterogeneity of the contact network will be at least as uncertain as disease incidence and prevalence. Thus, estimation of contact heterogeneity may be an important goal of the analysis. We note that previous work (e.g., [61]) has also discussed the potential use of sequence data to estimate contact heterogeneity.

## 5. Conclusions

 Contact heterogeneity is well known to have a strong effect on infectious disease dynamics. We have shown how the relationship between infectious disease dynamics and genealogies is similarly sensitive to the contact heterogeneity specified by a network. We have argued that direct knowledge of the tree of infections is likely needed in addition to sequence data for the accurate inference of prevalence from sequence data. Thus, it seems that understanding the structure of the contact networks for various diseases will be important for progress in phylodynamics.
